# Comparative Study of Corrosion Behaviors of WC-NiMo and WC-Co Cemented Carbides

**DOI:** 10.3390/ma16124480

**Published:** 2023-06-20

**Authors:** Nádia Alves Nery Balbino, Edmilson Otoni Corrêa, Danilo Roque Huanca, Flávio Amaury de Freitas Matos, Livio de Carvalho Valeriano

**Affiliations:** 1 Institute of Natural Resources, Federal University of Itajubá, Itajubá 37500-903, Brazil; 2 Institute of Mechanical Engineering, Federal University of Itajubá, Itajubá 37500-903, Brazil; ecotoni@unifei.edu.br (E.O.C.); flavio_eme@unifei.edu.br (F.A.d.F.M.); liviovaleriano@yahoo.com.br (L.d.C.V.); 3 Institute of Physics and Chemistry, Federal University of Itajubá, Itajubá 37500-903, Brazil; droqueh@unifei.edu.br

**Keywords:** WC-Ni cemented carbides, corrosion resistance, potentiodynamic polarization, electrochemical impedance spectroscopy, tungsten carbide

## Abstract

In this paper, the influence of a nickel binder metal and molybdenum carbide as an additional alloying element on the microstructure and corrosion behavior of WC-based cemented carbides, processed by conventional powder metallurgy, was studied, and a comparison with conventional cemented carbide (WC-Co) was carried out. The sintered alloys were characterized, before and after corrosive tests, by analyses using optical microscopy, scanning electron microscopy, energy dispersive X-ray spectroscopy, and X-ray diffraction. The corrosion resistance of the cemented carbides was investigated by open circuit potential, potentiodynamic polarization, and electrochemical impedance spectroscopy in a 3.5 wt.% NaCl solution. The WC-NiMo cemented carbides showed microstructures similar to those of WC-Co; however, pores and binder islands were observed in the microstructures. The corrosion tests showed promising results, the WC-NiMo cemented carbide showed superior corrosion resistance and higher passivation capacity than the WC-Co cemented carbide. The WC-NiMo alloy showed a higher $E_{OC}\;\left(\approx -0.18\;V\;vs.\;Ag|AgCl|{KCl}_{3mol/L}\right)$ than the WC-Co ($E_{OC}\approx -0.45\;V\;vs.\;Ag|AgCl|{KCl}_{3mol/L})$. The potentiodynamic polarization curves showed lower current density values throughout the potential range for the WC-NiMo alloy, and it was observed that E_corr_ was less negative $\left(\approx -0.416\;V\;vs.\;Ag|AgCl|{KCl}_{3mol/L}\right)$ than for WC-Co $(\approx -0.543\;V\;vs.\;V\;vs.\;Ag|AgCl|{KCl}_{3mol/L}$). The EIS analysis confirmed low rate corrosion of WC-NiMo associated with the formation of a passive thin layer. This alloy showed a higher *R_ct_* (1970.70 Ω).

## 1. Introduction

Cemented carbide is used to designate metal matrix composites comprising hard ceramic particles, normally tungsten carbide (WC), and a ductile binder matrix [1]. This material presents remarkable mechanical properties, has many potential applications, and is one of the most attractive powder metallurgy materials [2].

It is well known that cemented carbide has unique and exceptional properties, e.g., high hardness, high strength, and good wear resistance, which means that this material has many industrial applications, including for geoengineering equipment, cutting tools, drill bits and wear parts [3]. In addition to possessing an adequate combination of strength and hardness, cemented carbides that are used in the chemical, mining and petroleum industries also need to exhibit good corrosion resistance [3,4].

Cobalt (Co) is normally used for the binder phase given its excellent wetting, adhesion, and mechanical properties [5]. Nevertheless, given market price fluctuations, Co’s environmental toxicity, and the search for cemented carbides with greater corrosion resistance, researchers have been studying an alternative binder phase [6,7].

The alternatives with the greatest potential for replacing cobalt are other transition metals, such as Ni, Fe and Cr [8]. The authors of [9] studied the cemented carbide WC-AISI 304, of which the main constituents are Ni, Fe and Cr. According to this work, the hardness of cemented carbide was increased while maintaining the wear rates in the same order when compared to WC-Co. As demonstrated by [7,10,11,12], WC–Ni-based cemented carbides show better corrosion resistance and a relatively lower price. Nevertheless, the mechanical properties of WC-Co are relatively superior to those of WC-Ni cemented carbides, due to the lower wettability of Ni compared to Co [7,10,11,12]. To overcome this deficiency, the addition of carbides, such as molybdenum carbide (Mo_2_C), has been studied to strengthen WC-Ni alloys using solid solution techniques [13,14].

The corrosion behavior of cemented carbides is significantly influenced by their binder composition. It is well known that the corrosion resistance of cemented carbides is improved by using nickel instead of cobalt. Refractory carbides, such as molybdenum carbides, are also reported to have a positive influence on corrosion resistance, but their influence remains a relatively under-researched area [15,16].

Previous studies have demonstrated that WC-Ni cemented carbide hardness and abrasion resistance significantly increase with the addition of Mo and Mo_2_C [13,17,18,19]. Furthermore, these additions may enhance corrosion resistance in corrosive environments and improve cemented carbide passivation [5,20].

However, the addition of refractory carbides has limited solubility in the binder phase. Thus, they should only be a complement and should have their addition controlled [21]. According to several investigators [18,19,22,23], additions of Mo_2_C larger than 2 wt.% do not improve the mechanical properties of the cemented carbide. On the contrary, it may cause the reverse effect. In addition, it may affect the wetting ability of the binder phase on WC particles, weakening the bonding of the binder–carbide interface and provoking higher porosity and the formation of a molybdenum-rich M_7_C_3_ carbide. Additionally, 2 wt.% ensures that the solubility limit is not reached. This will lead to solid solution formation, reported to improve the mechanical properties of cemented carbides with Ni as a binder.

The objective of this study is, therefore, to evaluate the microstructure and corrosion behavior of WC-NiMo compared to WC-Co cemented carbides, processed by conventional powder metallurgy.

## 2. Materials and Methods

### 2.1. Preparing the Materials and Manufacturing the Alloys

The contents of the initial powders used in this study are listed in Table 1.

The cemented carbides were manufactured by conventional powder metallurgical technology. Powder of tungsten carbide (average particle size of 2.5 μm), carbonyl Ni (average particle size of 5 μm), and 99% pure molybdenum carbide (average particle size of 3 μm) were mixed with a ball-to-powder weight ratio of about 5:1. The conventional mixing was performed in a universal carbide-coated ball mill (whit carbide balls) at a horizontal rotation velocity of 50 rpm for 70 h. Heptane was added to the mixture to act as a process controlling agent. After mixing, 1.5 wt.% of paraffin was added to the mixtures to improve its compaction. Then, these mixtures were granulated and compacted into a stainless steel die at 140 MPa for 3 min. Then, the green compacts were pre-sintered in a pure hydrogen atmosphere, at 750 °C for 30 min. Finally, sintering was carried out in a high-vacuum atmosphere (2 to 6 × 10^−5^ bar) at 1.460 °C for 1 h to complete the metallurgical bonds between powder particles. The alloys were ground and polished with sandpaper and diamond paste.

### 2.2. Microstructures and Chemical Characterization

The microstructures and chemical characterization of the alloys were carried out before and after the corrosion tests (potentiodynamic polarization). Optical microscopy (OM) (Olympus, BX41M-LED, Tokyo, Japan) and scanning electron microscopy (SEM) (ZEISS, EVO MA 15, Jena, Germany) equipped with energy-dispersive X-ray spectroscopy (EDS) (Bruker, xFlash 360, Billerica, MA, USA) were performed on the polished surfaces to characterize the microstructure. The phase compositions of the alloys were detected using X-ray diffraction (XRD) (PANalytical, X’Pert PRO, Malvern, UK) at 40 kV, 40 mA, with Cu Kα (λ = 0.15406 Å) at a scanning step size of 0.02° in the scan range of 20–100°. Phase identification was carried out using the X’Pert HighScore Plus 3.0 software program and using crystallographic spreadsheets 43,380 (WC), 64,989 (Ni) and 76,942 (Co) from the ICSD (Inorganic Crystal Structure Database).

### 2.3. Electrochemical Measurements

The electrochemical study consisted of three main tests: (1) the open circuit potential (OCP), (2) the potentiodynamic polarization and (3) the EIS (electrochemical impedance spectroscopy). All experiments were performed with a PGSTAT302N Potentiostat unit (Autolab Metrohm, Utrecht, The Netherlands) in electrochemical cells with three-electrode arrangements using the cemented carbides as working electrodes with an exposure area of 28.3 mm^2^, a platinum sheet as the counter electrode, and a Ag|AgCl|KCl_3mol/L_ electrode as reference. The corrosive medium used was 3.5 wt.% NaCl aqueous solution, prepared with pro-analysis grade reagent and distilled water inside a Faraday cage at room temperature, 25 (± 2) °C, in an open air environment.

The OCP ${(E}_{OC})$ was monitored for 4 h. The potentiodynamic polarization measurements were performed over a potential scanning range from −500 to +1200 mV ${(E}_{OC})$, with a scan rate of 1 mV/s. EIS measurements were taken at open circuit potential, with a 10 mV (rms) sinusoidal perturbation from 10 kHz to 10 mHz (frequency range) and at ten points per frequency decade. The potentiodynamic polarization and EIS measurements were carried out 1 h after immersion, which stabilized the $E_{OC}$ values with a range of ±0.01 V. All measurements were repeated three times, and the results showed good repeatability. All corresponding parameters were calculated and simulated directly using the accompanying software (NOVA 2.1) program from the Autolab unit.

## 3. Results

### 3.1. Microstructural and Chemical Characterization before Corrosion

**Figure 1:**
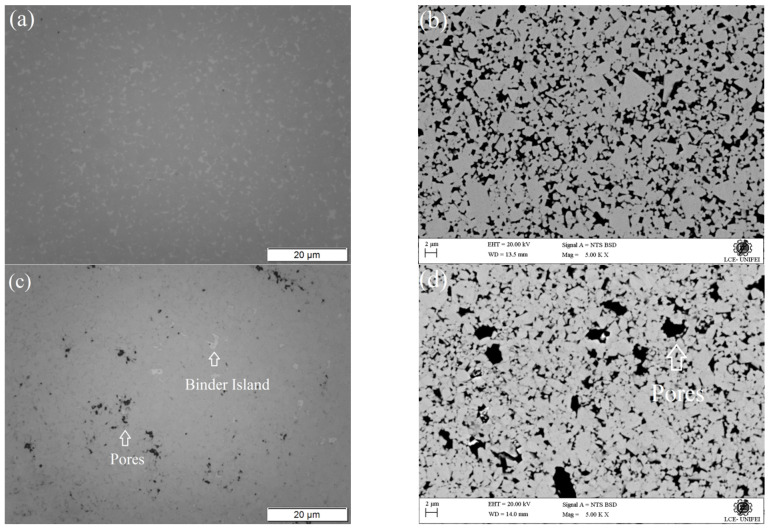
(**a**) OM micrograph of the WC-Co alloy. (**b**) SEM micrograph (BSE mode) of the WC-Co alloy. (**c**) OM micrograph of the WC-NiMo alloy. (**d**) SEM micrograph (BSE mode) of the WC-NiMo alloy. Before potentiodynamic polarization in a 3.5 wt.% NaCl solution.

Figure 1 shows the OM and SEM morphology observations for the WC-Co and WC-NiMo alloys before the corrosion tests. Homogeneous distribution of the binder phase (lighter spots) was observed in the OM micrograph (Figure 1a), which occurred due to the good wettability of Co, i.e., Co showed an ability to diffuse throughout the WC grains during liquid phase sintering. The SEM micrograph (Figure 1b) shows a heterogeneous WC grain microstructure and distribution (bright phase) surrounded by a Co metal matrix (dark phase). There was no significant amount of precipitated graphite or brittle η-phases in the microstructure.

One can see in Figure 1c,d that the microstructure is similar to the WC-Co alloy. No significant amount of precipitated graphite or brittle phases in the microstructure were observed. Furthermore, the presence of undissolved Mo_2_C particles was not noted. These observations suggest that the sintering parameters and the Ni, Mo and C contents were adequate.

However, the presence of binder islands (accumulation of the binder phase in some regions) and pores uniformly distributed in the microstructure were detected. According to [14], these islands of binder phase show a lower uniformity regarding the binder distribution in the microstructure in comparison with that observed in the WC-Co. This may be due to the low wetting and WC dissolution rate of the Ni binder when compared with the Co binder, which led to a deficient spreading of the Ni liquid phase between the grains as it dissolves WC. Moreover, the lower chemical homogeneity in the prepared compacts, as a result of insufficient mixing conditions, may also contribute significantly to this uneven distribution of the binder metal. This microstructure is quite similar to those observed by [19,22].

During the sintering process, the viscous flow of the binder phase plays a major role in the densification behavior of the alloy. As Co has a higher wettability compared to WC than Ni, alloys containing Co have higher densification, that is, lower pore volume. Furthermore, Mo_2_C preferentially dissolves in the binder phase during the sintering process, which may cause a negative influence on the flow ability of the liquid phase. Therefore, the pores in the alloys cannot be filled by the liquid phase during the sintering process, which leads to the presence of pores and decline in relative density [5].

**Figure 2:**
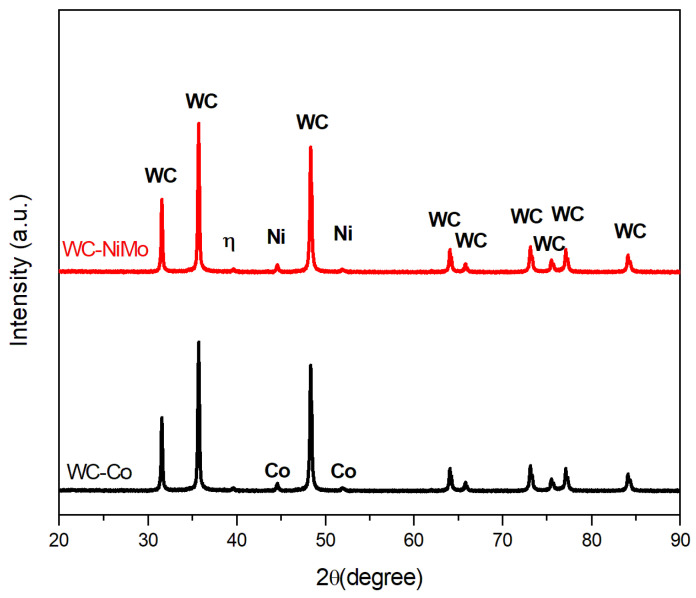
Diffractograms of WC-Co and WC-NiMo alloys.

XRD analyses of WC-Co and WC-NiMo are shown in Figure 2. Only WC and the binder phase (Co and Ni) were identified in both composites, which are the predominant phases. No Mo_2_C or Mo peaks were detected due to the small additions. Furthermore, no graphite peaks were observed, confirming that there was no significant lack or excess of carbon in the alloy system during sintering. Peaks of a new phase were observed, as shown in Figure 2, possibly indicating a small formation of brittle η-phases, but these peaks were very tiny and could not be confirmed by comparing the peak characteristics [3].

The chemical quantifications of the alloys, obtained via EDS analysis, are shown in Table 2. These were within the expected levels given the contents and compositions of the raw materials, indicating that there were no considerable losses in the cemented carbide production process.

### 3.2. Open Circuit Potential

**Figure 3:**
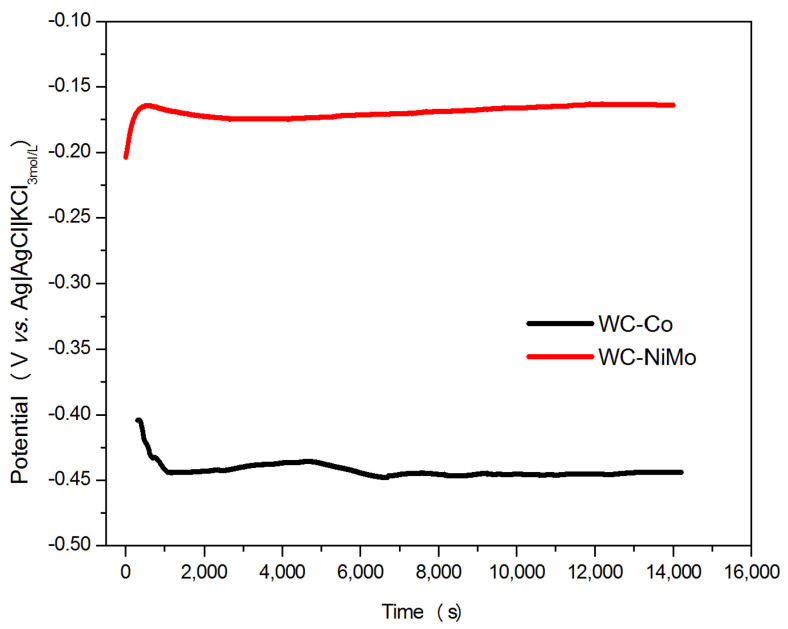
The open circuit potential ($E_{OC}$) curves for the WC-Co and WC-NiMo alloys in a 3.5 wt.% NaCl solution, at room temperature for approximately 4 h.

The $E_{OC}$ versus time curves for the WC-Co and WC-NiMo alloys are presented in Figure 3. For the case of WC-Co, the initial shift of $E_{OC}$ from about −0.40 to −0.45 V vs. Ag|AgCl|KCl_3mol/L_ during the first 1000 s may indicate corrosive attacks of the sample surface by the solution. Inversely, an increasing behavior in the $E_{OC}$ value in time was observed for the WC-NiMo alloy, which suggests the formation of some passivation films on the surface during the first 500 s approximately, after which it remains almost constant and acts as a barrier against alloy dissolution. The anti-corrosion characteristic of this alloy is also seen through the less negative position of $E_{OC}\left(\approx -0.18\;V\;vs.\;Ag|AgCl|{KCl}_{3mol/L}\right)$ if compared with the WC-Co ($E_{OC}\approx -0.45\;V\;vs.\;Ag|AgCl|{KCl}_{3mol/L})$ alloy [20]. The persistent variation in the $E_{OC}$ values with immersion time for the WC-Co alloy may be associated with the lowest thermodynamic stability of its surface against corrosion.

### 3.3. Potentiodynamic Polarization Curves

**Figure 4:**
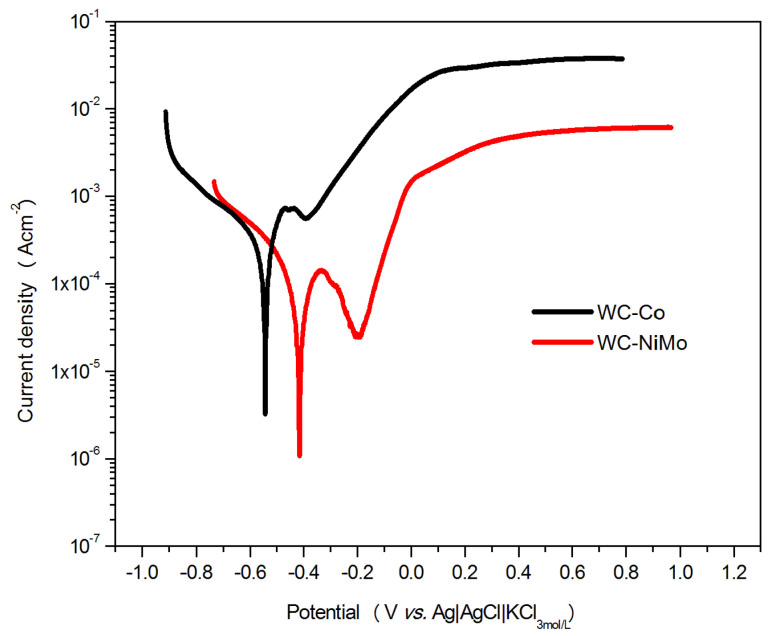
The potentiodynamic polarization curves of the WC-Co and WC-NiMo alloys in a 3.5 wt.% NaCl solution, at room temperature.

The polarization curves for the WC-Co and WC-NiMo alloys are shown in Figure 4. The curves exhibit similar behaviors. In the cathode branch, for both materials, one can see a slight plateau, which was attributed to the reduction of oxygen (Equation (1)) [24].

(1) $$O_{2}+{2H}_{2}O_{\left(l\right)}+4e^{-}\to 4{OH}_{\left(aq\right)}^{-}$$

In the branch of anodic polarization, with increasing potential (${E>E}_{corr})$, the current densities increased exponentially, indicating an active dissolution corrosion mechanism controlled by the binder phase [25]. One can see that the current densities arise from the binder phase oxidation on the surface, due to the standard redox potentials for Co oxidation (Eo=−518 mV) and Ni oxidation $\left(Eo=-491\;mV\right)$ [20,26].

The critical current densities were reached with increasing polarization, and then, the current densities quickly decreased, thus obtaining the pseudopassive regions. At higher potentials, the current density rises again, thus reaching the transpassive region, where the increase in current density can also be related to WC oxidation [27,28].

Although the WC-Co pseudopassive behavior was similar to the passive behavior, it was different from the general passivity. The magnitude of the current density remained high (around $550\;µA{cm}^{-2}$) relative to the typical values for passive current densities (below $10\;µA{cm}^{-2}$) [7]. This behavior is explained by the increased Co diffusion path length. After the dissolution of the Co present at the surface, the current flow was limited because Co has to diffuse throughout the remaining porous tungsten carbide skeleton [16].

Lower current density values were observed throughout the potential range for the WC-NiMo alloy, showing nobler behavior relative to the WC-Co alloy. The current density dropped sharply in the pseudopassive region to a minimum around 25 $µA{cm}^{-2}$, very close to the typical value for passive current densities (below $10\;µA{cm}^{-2}$) [7]. The pseudopassive region was expanded for the WC-NiMo alloy (−0.33 to −0.18 V vs. Ag|AgCl|KCl_3mol/L_), compared to the WC-Co alloy (−0.47 to −0.38 V vs. Ag|AgCl|KCl_3mol/L_), indicating that the binder composition can enhance the passivation behavior of the cemented carbide. Furthermore, the transpassive region of the WC-NiMo alloy had lower current densities values, i.e., nobler behavior.

According to [29], Mo atoms can form a solid solution with the binder phase, which can strengthen the binder phase and improve the anti-corrosive ability of the carbide as a whole. It was reported by [20] that Mo existing on the surface can be converted to oxides in the passive region, and this may have a large impact on the passivation behavior. Although the pseudopassive current density is still slightly greater than 10 µA, the drop in current density is caused by a protective oxide film, with a similar process observed in passivation, which is different from what happens for the WC-Co alloy.

The electrochemical parameters that describe the potentiodynamic curves of the cemented carbides, determined according to the Tafel extrapolation technique, are listed in Table 3. The corrosion potential $\left(E_{corr}\right)$ indicates the thermodynamic stability of the tested alloys under electrochemical corrosion conditions. The more negative the $E_{corr}$, the more noble the material [30]. Thus, comparing the $E_{corr}$ for both alloys, it was observed that $E_{corr}$ for WC-NiMo is less negative $\left(\approx -0.416\;V\;vs.\;Ag|AgCl|{KCl}_{3mol/L}\right)$ than for WC-Co $\left(\approx -0.543\;V\;vs.\;V\;vs.\;Ag|AgCl|{KCl}_{3mol/L}\right)$, thus showing its noble nature with greater resistance to corrosion. As already discussed by the open circuit potential analysis, this positive potential shift is associated with the spontaneous formation of a passive film [26].

Concerning $i_{corr}$, it represents the kinetics of material corrosion and is proportional to the corrosion rate [25]. Consequently, the greater corrosion resistance of the WC-NiMo alloy, if compared with WC-Co, is the reason the $i_{corr}$ is lower, i.e., low charge exchange through the surface–liquid interface occurs. This is also the reason that the passive current density ($i_{pass})$ is also lower for the case of the WC-NiMo alloy. These results show the significant improvements in the corrosive behavior of WC-NiMo if compared to the WC-Co alloy. Comparable results were earlier reported in the literature [7,20,26,31].

### 3.4. Electrochemical Impedance Spectroscopy

**Figure 5:**
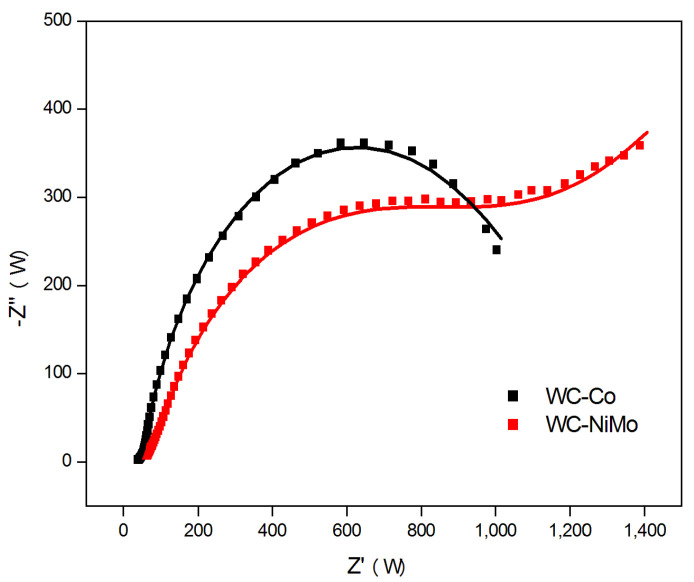
Nyquist diagrams of experimental data points and fitting curves showing the impedance spectra for the WC-Co and WC-NiMo alloys in a 3.5 wt.% NaCl solution.

**Figure 6:**
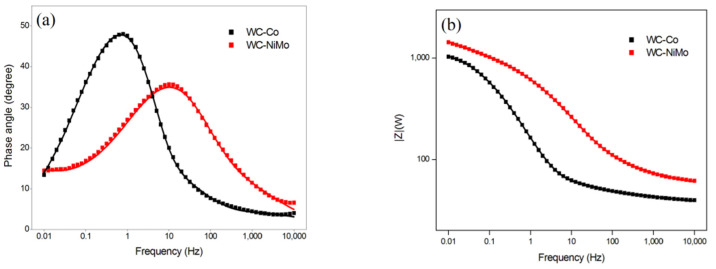
Bode diagrams of experimental data points and fitting curves showing the impedance spectra for the WC-Co and WC-NiMo alloys in a 3.5 wt.% NaCl solution. (**a**) Phase angle. (**b**) Total impedance.

The Nyquist and Bode diagrams for the WC-Co and WC-NiMo alloys are plotted in Figure 5 and Figure 6, respectively.

The size of the semicircle diameter is associated with the magnitude of the charge transfer $\left(R_{ct}\right)$ of the alloy–liquid system, thus reflecting the rate of electrochemical reactions. Larger semicircle diameters correspond to low corrosion rates associated with low charge transfer through the alloy–liquid interface [32]. For both alloys, the Nyquist plot shows a semicircle feature in the middle- and high-frequency regions; however, for the WC-NiMo alloy, in addition to this semicircle, the presence of a straight line in the low-frequency region suggests the existence of impedance diffusion that is adequately described by a Warburg element. This impedance diffusion is related to difficulties in mass transport through an oxide product. The absence of this straight line in Figure 5 indicates that in the WC-Co alloy, the reaction mechanisms are controlled predominately by charge transfer through the double layer, while for the WC-NiMo alloy, the ion diffusion reaction predominates [32,33].

**Figure 7:**
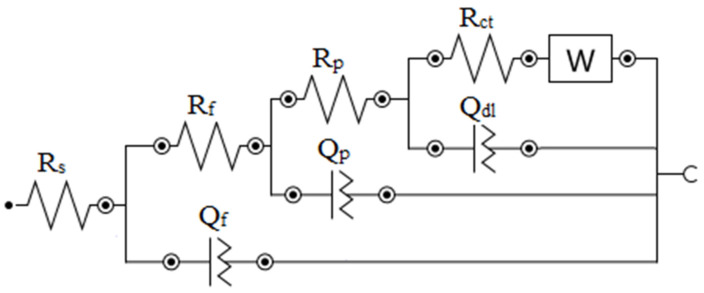
The equivalent EIS circuit for the WC-NiMo alloys in a 3.5 wt.% NaCl solution. In the case of WC-Co, the Warburg element is disregarded.

Conversely, the non-symmetric bode phase plot (Figure 6) of both alloys suggests that one semicircle is the result of the overlap of various minor semicircles linked to the different elements present on the alloy surface (pores, oxide film, and double layer). Thus, these impedance curves were well fit by the proposed equivalent circuits seen in Figure 7, where the Warburg element (W) was included to describe the ionic diffusion-controlled reaction mechanism to adequately describe the impedance behavior of the WC-NiMo alloy, while for the WC-Co alloy, it was disregarded. In this circuit, $R_{s}$, $R_{f},R_{p}$ and $R_{ct}$ represent the resistance to a corrosive solution (uncompensated resistance), product films, pores, and charge transfer, respectively. $Q_{f}$, $Q_{p}$ and $Q_{dl}$ represent the constant phase element (CPE) of passive films, pores and the double charge layer, respectively. The CPE represents the non-ideal frequency-dependent capacitance associated with the surface heterogeneities, non-planar interfaces and irregularities [34]. Under this condition, the impedance can be determined via Equation (2) [35]:

(2) $$Z_{CPE}=\frac{1}{{Q_{CPE}(j\omega )}^{n}}$$

where ω is the angular frequency, and n (0≤n≤1) and *Q* are parameters that are independent of the frequency. When n=1, *Q* corresponds to an ideal capacitor; otherwise, *Q* characterizes the non-uniform charge distribution along the electrode/solution interface due to surface heterogeneities [36].

This proposed circuit, shown in Figure 7, offers the most coherent physical interpretations of the system, as depicted in Figure 5 and Figure 6, where the fitting IES curves appear as solid lines, and the fitting values of the different electrical elements are summarized in Table 4.

$R_{ct}$ is an important parameter for this technique because it is a measure of the corrosion rate. The higher the $R_{ct}$, the higher the resistance to corrosion [37]. According to these results (Table 4), although with the naked eye the semicircle feature corresponding to the WC-Co alloys seems to be greater, the fitting result indicates that for this case, $R_{ct}$ equals 1222.40 Ω, while for the WC-NiMo alloy, it is 1970.70 Ω. This fact confirms, again, the primary observations by open circuit potential analysis and dynamic potentiometry, i.e., a low rate of corrosion of WC-NiMo is associated with the formation of a passive thin layer that acts as a corrosion protective barrier. Concerning $R_{s}$, it was also higher for the WC-NiMo alloy and may be influenced by the resistance associated with the corrosion product films [34].

The results concerning oxide film formation reveal that although it is formed in both alloys, the larger value of $R_{f}$ (53.03 Ω) for the case of WC-NiMo shows that it is more stable than in WC-Co ($R_{f}$ = 13.10 Ω) and was formed even inside the pores or pits, as can be seen by the larger value of $R_{p}$ (295.49 Ω) for this latter alloy. Although the corrosion test describes that WC-NiMo has a more stable surface, the IES analysis reveals a lower $n_{dl}$ (0.21) associated with the formation of deep pores, in the order of 10 µm, on the surface of the as-sintered alloys, as observed in Figure 1. This singular feature also explains why the Bode phase plot of this alloy appears to have a broader phase angle peak with a lower height compared to the WC-Co alloy, while its phase peak position reveals its higher total impedance magnitude.

Corrosion mechanisms can be better interpreted by equivalent circuits. Nevertheless, given the complex microstructure of cemented carbides and the presence of pores, it is not trivial to analyze the data and to extract useful information. Microstructural information must complement the analysis of impedance data. The analysis of different techniques together is important for a better understanding of corrosive behavior [36].

### 3.5. Microstructural and Chemical Characterization after Corrosion

**Figure 8:**
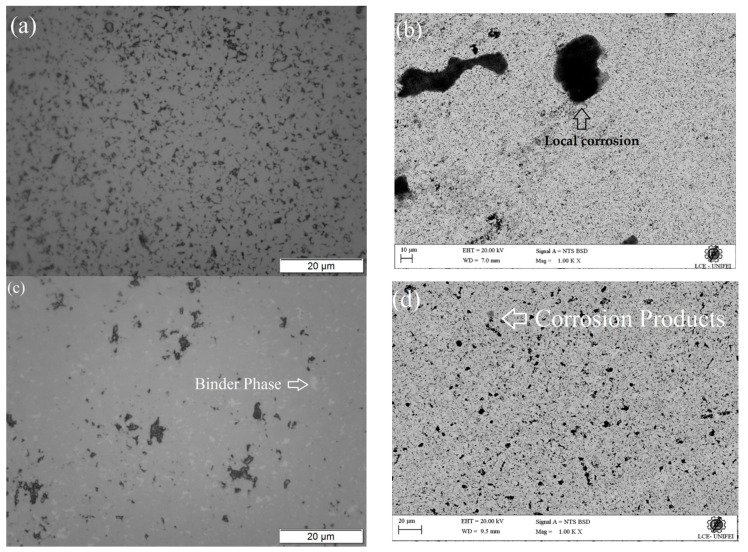
(**a**) OM micrograph of the WC-Co alloy. (**b**) SEM micrograph (BSE mode) of the WC-Co alloy. (**c**) OM micrograph of the WC-NiMo alloy. (**d**) SEM micrograph (BSE mode) of the WC-NiMo alloy. After potentiodynamic polarization in a 3.5 wt.% NaCl solution.

Figure 8 shows the OM and SEM morphologies of the WC-Co and WC-NiMo alloys after potentiodynamic polarization. In the OM micrograph (Figure 8a), one can note the absence of metallic Co and the exposed WC grains on the surfaces of the alloy, indicating a severe dissolution of the Co binder phase. This is evident when comparing Figure 8a with Figure 1a. As shown in Figure 8b, some regions present localized corrosion. Preferential dissolution of the Co binder through galvanic coupling between the WC grains and Co binder occurs with the corrosion process, leaving some pores on the surface. Subsequently, corrosion of the Co binder phase continues to dissolve, and the pores are increased, causing desquamation of the WC grains. Thereafter, electrolyte penetration in the corroded areas takes place, which can lead to local corrosion [29,38]. Furthermore, small coating defects, mainly around the WC particles, together with galvanic effects, assist in the accelerated dissolution of the Co phase at specific sites, generating deep attacks in these areas [24]. It is believed that the oxidation of the metal is the main reaction in the undermined regions, since the reduction of $O_{2}$ is not expected to occur due to its limited access to these internal locations. The dissolution of Co can be accelerated by the acidification of the internal solution due to the hydrolysis of metallic cations [39].

The binder phase can still be observed (light spots) in Figure 8c, unlike the WC-Co alloy, suggesting that severe dissolution of the binder phase did not happen in the WC-NiMo alloy. These results corroborate the existing literature [5,17,20]. Figure 8d shows that the localized corrosion was minimized, with only small areas of accumulated corroded products. According to [26], the presence of Mo significantly improved localized corrosion resistance for the cemented carbides. Furthermore, Figure 8c shows that small accumulated corroded products occurred preferentially in the binder islands and in the pores of the microstructure. The binder islands can act as a starting point for corrosion, because they are the most exposed phase, and the pores may lead to faster electrolyte penetration into the microstructure, causing localized attacks [40,41].

**Figure 9:**
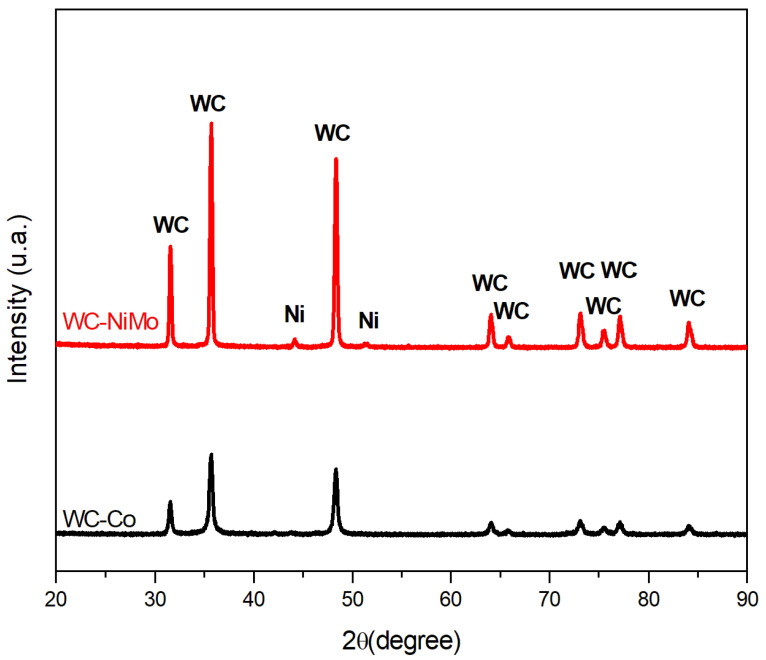
Diffractograms of the WC-Co and WC-NiMo alloys after potentiodynamic polarization in 3.5 wt.% NaCl solution.

The XRD analysis for the WC-Co and WC-NiMo alloys after potentiodynamic polarization are shown in Figure 9. In both composites, the WC continues to be seen, which is the predominant phase. However, the Co peaks are no longer seen, while the Ni peaks continue to be present. Thus, these results also indicate severe dissolution of the Co binder phase, which is coherent with those earlier reported in the literature regarding WC-Co cemented carbide corrosion [42,43,44]. Furthermore, the significant reduction in WC peak intensity for the WC-Co alloy is linked to the intense corrosion that occurred in this alloy.

Severe dissolution of the Co binder phase was also confirmed by EDS analysis made after exposure of the alloys to the aqueous solution. The chemical quantification of both the WC-Co and WC-NiMo alloys is summarized in Table 5. Comparing these results with that reported in Table 2, the abrupt reduction in Co is noticeable (from 8.86 to 0.23% of concentration), is compatible with the severe dissolution of the Co binder phase, and the superior corrosion resistance of the WC-NiMo alloy in which Ni content (≈7.78%) remains is almost constant within the experimental error. Concerning the Co binder phase, it is corroded to form ${Co}_{3}O_{4}$ and ${Co(OH)}_{2}$, which are easily dissolved into the solution, and the WC particles are released, which slowly react to form ${WO}_{3}$ [29,45]. In addition to Mo, although no peaks of it are detected after the dissolution experiment of the WC-NiMo alloy, its absence seems to be associated with a low concentration below the detection resolution. However, according to the literature [5,29], this element in the binder phase can be oxidized to form $MoO_{3}$ and then adheres to the surface of cemented carbide to isolate the corrosion solution. This fact is in concordance with the electrochemical techniques results, which suggest the formation of a stable pseudopassive film for the WC-NiMo alloy.

## 4. Conclusions

This paper shows a comparative study of the corrosion behavior of WC-NiMo and WC-Co cemented carbides in a 3.5 wt.% NaCl solution. The results suggest that the WC-NiMo alloy has higher corrosion resistance and greater passivation capacity in the NaCl solution compared to the WC-Co alloy.

The linear potentiodynamic polarization tests showed pseudopassive behavior for both cemented carbides. Active dissolution of the binder phase was observed for the WC-Co alloy, and the pseudopassive behavior was attributed to the increased length of the Co diffusion path. Noble behavior was observed in the entire analyzed frequency range for the WC-NiMo alloy, expanded pseudopassive regions, and considerably lower current densities, with the minimum values very close to passive levels. The results of the potentiodynamic polarization measurements generally agree with those observed in the $E_{OC}$ curves, EIS spectra, and the microstructural and chemical characterization tests after corrosion.

The results from the different techniques were coherent and showed the need for using various methods, which should be complemented with microstructural information for a better understanding of the electrochemical behavior of the cemented carbides.

## Figures and Tables

**Table 1:** The nominal composition of alloys (wt.%).

Cemented Carbide	WC	Co	Ni	Mo_2_C
WC-Co	90	10	-	-
WC-NiMo	90	-	8	2

**Table 2:** EDS results for the polished surface for the WC-Co and WC-NiMo alloys.

Alloy	Chemical Composition (%)					
	W	C	O	Co	Ni	Mo
WC-Co	80.69	9.28	1.17	8.86	-	-
WC-NiMo	80.64	9.34	1.07	-	7.03	1.93

**Table 3:** The corresponding electrochemical parameters for the WC-Co and the WC-NiMo alloys in a 3.5 wt.% NaCl solution, at room temperature, determined according to the Tafel extrapolation technique.

Alloy	$E_{corr}$ $\left(V\;vs.Ag|AgCl|{KCl}_{3mol/L}\right)$	$i_{corr}$ $\left(µA{cm}^{-2}\right)$	$i_{crit}$ $\left(µA{cm}^{-2}\right)$	$i_{pass}$ $\left(µA{cm}^{-2}\right)$
WC-Co	−0.543	1190.40	725.74	556.43
WC-NiMo	−0.416	129.68	142.34	24.80

**Table 4:** Equivalent circuit parameter values for EIS data for WC-Co and WC-NiMo alloys in a 3.5 wt.% NaCl solution.

	WC-Co	WC-NiMo
$R_{s}(Ω)$	35.75	56.02
$R_{f}(Ω)$	13.10	53.03
$Q_{f}({µ\Omega }^{-1}s^{n})$	766.18	155.76
$n_{f}$	0.50	0.61
$R_{p}(\Omega )$	41.93	295.49
$Q_{p}({µ\Omega }^{-1}s^{n})$	842.17	97.60
$n_{p}$	0.75	0.72
$R_{ct}(\Omega )$	1222.40	1970.70
$Q_{dl}({µ\Omega }^{-1}s^{n})$	294.44	942.37
$n_{dl}$	0.90	0.21
$W({µ\Omega }^{-1}s^{0.5})$	-	3973.30

**Table 5:** EDS results for the surface for the WC-Co and WC-NiMo alloys, after potentiodynamic polarization in 3.5 wt.% NaCl solution.

Alloy	Chemical Composition (%)					
	W	C	O	Co	Ni	Mo
WC-Co	90.34	8.39	1.01	0.23	-	-
WC-NiMo	81.65	9.40	1.18	-	7.78	0.00

## Data Availability

Not applicable.
